# Myxedema Coma and the Heart: Would You Miss the Signs?

**DOI:** 10.7759/cureus.45164

**Published:** 2023-09-13

**Authors:** Juan I Vazquez-Fuster, Giovanni Rivera, Sonia I Vicenty-Rivera, Francisco Merced

**Affiliations:** 1 Cardiology, Veteran Affairs Caribbean Healthcare System, San Juan, PRI; 2 Internal Medicine, Veteran Affairs Caribbean Healthcare System, San Juan, PRI

**Keywords:** cardiovascular intensive care unit, hypothyroid pericardial effusion, hypothyroid myxedema coma, focused cardiac ultrasound, bradyarrhythmias

## Abstract

Myxedema Coma (MC) is a life-threatening medical emergency that occurs as a severe complication of untreated or poorly managed hypothyroidism. Prompt diagnosis is crucial as the condition can rapidly deteriorate and lead to life-threatening complications. Timely treatment of myxedema coma with intravenous levothyroxine is the cornerstone of treatment, along with glucocorticoids to support adrenal function. This condition is associated with cardiovascular manifestations that contribute to its high mortality rate. The heart in hypothyroidism typically shows reversible dysfunction that can be corrected with hormonal supplementation, and in some cases, requires inotropic and aminergic support. This case involves a patient who was admitted to the intensive care unit with suspected MC, and necessitated life-saving hormonal and cardiovascular support to manage the condition.

## Introduction

MC is a severe form of intracellular T3 hormone depletion which can result in respiratory, metabolic, neurologic, and cardiac dysfunction. It is considered an endocrine emergency with mortality rates that range from 25 to 65% [1]. Diagnosis is based on clinical suspicion and patients more commonly present with altered sensorium, hypothermia, hyponatremia and/or hypercapnia. Cardiovascular manifestations are considered the main cause of mortality in this disease, and they include bradycardia, hypotension, congestive heart failure, pericardial effusions, conduction defects and arrhythmias. The heart’s reversible behavior in hypothyroidism can be corrected with hormone supplementation, and in some cases, may require inotropic and aminergic support [2]. This case presents a patient in which MC was high in the initial differential who eventually required life-saving hormonal and cardiovascular support.

## Case presentation

**Figure 1:**
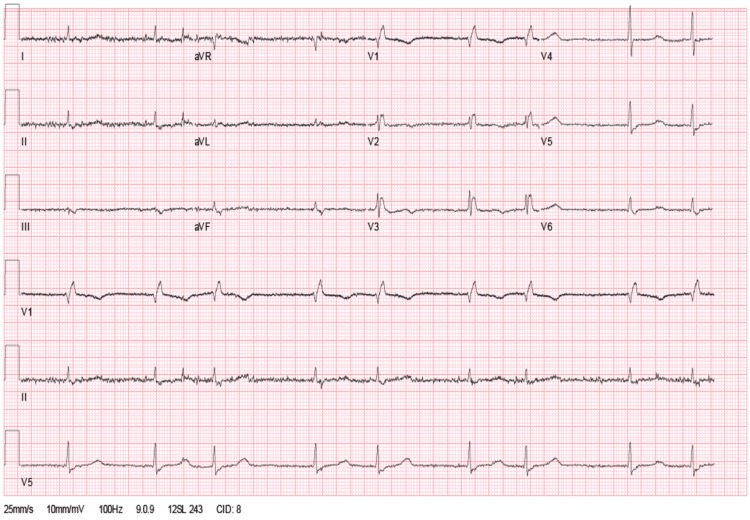
Electrocardiogram showing atrial fibrillation with slow ventricular response and low voltage in the limb leads

An 88-year-old male with a medical history of Alzheimer's dementia, atrial fibrillation, and hypothyroidism was brought to the emergency department due to a two-day history of hypoactivity. Vital signs on arrival showed hypotension (95/24 mmHg), bradycardia (42 beats per minute), and hypothermia (34.1° Celsius). Physical examination was remarkable for a hypoactive patient responsive to painful stimuli and diminished deep tendon reflexes. The electrocardiogram showed atrial fibrillation with slow ventricular response, low voltage in the limb leads, and no acute ST segment changes or T wave inversions (Figure 1). Point of care ultrasound was negative for a hemodynamically significant pericardial effusion (Video 1). Laboratory findings were remarkable for a thyroid stimulating hormone (TSH) of 217 mIU/L, T3 of 19.5 ng/dl, and T4 of 0.117 ng/dl. A head computed tomography came back negative for any acute intracranial pathology.

**Video 1:** Echocardiogram in the parasternal long axis view showing mild left ventricular systolic dysfunction and no pericardial effusion.

Myxedema coma was high in the differential diagnosis, reason why a loading dose of intravenous levothyroxine was provided. Hydrocortisone was given prior to levothyroxine administration for suspected concomitant adrenal insufficiency. Dopamine and norepinephrine infusions provided hemodynamic improvement. After five days of hormonal supplementation and passive rewarming, hypoactivity resolved, vasoactive medications were discontinued, and he was transferred to the internal medicine ward for continuity of care. Follow-up TSH improved to 15 mIU/L.

## Discussion

Myxedema coma (MC) is a rare, life-threatening complication of severe hypothyroidism. While it can occur in patients with undiagnosed or undertreated hypothyroidism, it is more commonly seen in elderly patients with a history of hypothyroidism who have failed to comply with their medications or have experienced acute illness or trauma [2]. Our patient was a nursing home resident due to underlying Alzheimer's dementia, who was completely dependent on his daily activities. Upon discussion with nursing home staff, the patient was not cooperating in taking his oral supplementation of Levothyroxine for several months, the reason why he arrived with this endocrine emergency. 

The hallmark feature of myxedema coma is a profound depression of metabolism, leading to decreased cardiac output, peripheral vasoconstriction, and bradycardia. Cardiovascular complications of myxedema coma include hypotension, bradycardia, pericardial effusion, decreased cardiac output, and decreased left ventricular systolic function. These complications can result in organ hypoperfusion, leading to multisystem organ failure. Cardiovascular collapse is the most common cause of death in myxedema coma, accounting for up to 50% of fatalities [2] and bradycardia is a known predictor of increased mortality [3]. MC is missed in 50% of patients arriving at the emergency room (ER) [4]. Thus, early recognition and management of cardiovascular dysfunction are crucial for preventing morbidity and mortality.

Hemodynamic stabilization should be the initial priority in the management of patients with MC. Patients with hypotension and shock require intravenous fluid resuscitation with isotonic saline, followed by vasopressor support with norepinephrine or dopamine if needed [2]. Bradycardia should be managed with atropine or temporary pacing, depending on the severity of symptoms. Our patient arrived remarkably hypotensive and bradycardic. Fortunately, norepinephrine and dopamine infusions provided adequate hemodynamic support and did not require temporary pacemaker implantation. His point of care ultrasound showed a small pericardial effusion with no findings to suggest cardiac tamponade. Pericardial effusion and tamponade, which are frequently observed, should be emphasized as they may necessitate emergent pericardiocentesis [1].

Hormone replacement therapy with intravenous levothyroxine is the mainstay of treatment in myxedema coma. A loading dose of 300 to 500 mcg of levothyroxine is recommended, followed by a maintenance dose of 50 to 100 mcg/day. Corticosteroid therapy may be considered in patients with suspected adrenal insufficiency or severe hypotension unresponsive to fluid resuscitation and vasopressor support [5]. This was the management provided to our patient and after five days of hormonal supplementation, hypoactivity resolved, vasoactive medications were discontinued and follow up TSH was 15 mIU/L.

## Conclusions

Myxedema coma is a rare complication of severe hypothyroidism, with cardiovascular complications being the most common cause of morbidity and mortality. Prompt recognition and management of cardiovascular dysfunction are crucial for improving outcomes. Aggressive supportive care, including hemodynamic stabilization and hormone replacement therapy, are essential components of management in these patients. Healthcare providers should be vigilant in monitoring and managing cardiovascular complications in patients with myxedema coma to reduce the risk of poor outcomes in this life-threatening emergency.
